# Glia-Mediated Regenerative Response Following Acute Excitotoxic Damage in the Postnatal Squamate Retina

**DOI:** 10.3389/fcell.2020.00406

**Published:** 2020-05-28

**Authors:** Julia Eymann, Nicolas Di-Poï

**Affiliations:** Research Program in Developmental Biology, Institute of Biotechnology, University of Helsinki, Helsinki, Finland

**Keywords:** central retina, retinal margin, regeneration, squamate, NMDA, Müller glia

## Abstract

The retina is a complex tissue responsible for both detection and primary processing of visual stimuli. Although all vertebrate retinas share a similar, multi-layered organization, the ability to regenerate individual retinal cells varies tremendously, being extremely limited in mammals and birds when compared to anamniotes such as fish and amphibians. However, little is yet known about damage response and regeneration of retinal tissues in “non-classical” squamate reptiles (lizards, snakes), which occupy a key phylogenetic position within amniotes and exhibit unique regenerative features in many tissues. Here, we address this gap by establishing and characterizing a model of excitotoxic retinal damage in bearded dragon lizard (*Pogona vitticeps*). We particularly focus on identifying, at the cellular and molecular level, a putative endogenous cellular source for retinal regeneration, as diverse self-repair strategies have been characterized in vertebrates using a variety of retinal injury and transgenic models. Our findings reveal for the first time that squamates hold the potential for postnatal retinal regeneration following acute injury. Although no changes occur in the activity of physiologically active progenitors recently identified at the peripheral retinal margin of bearded dragon, two distinct successive populations of proliferating cells at central retina respond to neurotoxin treatment. Following an initial microglia response, a second source of proliferating cells exhibit common hallmarks of vertebrate Müller glia (MG) activation, including cell cycle re-entry, dedifferentiation into a progenitor-like phenotype, and re-expression of proneural markers. The observed lizard glial responses, although not as substantial as in anamniotes, appear more robust than the absent or neonatal-limited regeneration reported without exogenous stimulation in other amniotes. Altogether, these results help to complete our evolutionary understanding of regenerative potential of the vertebrate retina, and further highlight the major importance of glial cells in retinal regeneration. Furthermore, our work offers a new powerful vertebrate model to elucidate the developmental and evolutionary bases of retinal regeneration within amniotes. Such new understanding of self-repair mechanisms in non-classical species endowed with regenerative properties may help designing therapeutic strategies for vertebrate retinal diseases.

## Introduction

The retina represents an essential part of the visual sensory system responsible for both detection and primary processing of visual stimuli. Impaired vision due to retinal disease or injury is ultimately life-threatening to many vertebrates, and dysfunction or death of retinal neurons are among the most common human ocular problems worldwide. Therefore, many efforts have been made to better understand and activate the intrinsic regenerative capacity of the retina, using a wide range of injury paradigms in various vertebrate species with different regenerative capacities (Jeon and Oh, 2015; Ail and Perron, 2017). In particular, non-mammalian organisms such as fish and amphibians have emerged as central model systems for studying retinal regeneration (Amato et al., 2004; Stenkamp, 2007; Alunni and Bally-Cuif, 2016; Hamon et al., 2016; Ail and Perron, 2017), due to their ability to regenerate numerous tissues in contrast to conventional experimental models such as mice and rat.

All vertebrate retinas share a similar, highly complex layered structure (Supplementary Figure S1A; Baden et al., 2020), comprising three nuclear layers (ONL, outer nuclear layer; INL, inner nuclear layer; GCL, ganglion cell layer) separated by two plexiform layers (OPL, outer plexiform layer; IPL, inner plexiform layer). Major cell types include photoreceptor cells in the ONL, amacrine cells, bipolar cells, horizontal cells as well as Müller glia (MG) in the INL; and finally ganglion cells and displaced amacrine cells in the GCL (Supplementary Figure S1A; Baden et al., 2020). Whereas cell bodies are located in the three nuclear layers, axonal and dendritic processes form a network of synapses in the plexiform layers. Importantly, all distinct neuronal cell types but also MG, the major glial cell type in the retina, derive developmentally from the same pool of multipotent retinal progenitor cells in vertebrate models such as rat (Turner and Cepko, 1987), mouse (Turner et al., 1990), *Xenopus* (Holt et al., 1988; Wetts and Fraser, 1988), zebrafish (Stenkamp, 2007), and chicken (Fekete et al., 1994). This common embryonic origin has suggested that endogenous stem/progenitor cells could be excellent candidates for retinal regeneration, through multi-lineage differentiation that would be integrated into injured or diseased retina. Based on various injury and/or transgenic models, different cellular sources for retinal regeneration have since been characterized in vertebrate species, including the circumferential marginal zone (CMZ) at the peripheral edge of the retina, the retinal pigmented epithelium (RPE), the ciliary body (CB) epithelium, and MG. Particularly, the retina of adult fish and amphibians exhibits unique regeneration features, ranging from full restoration of the entire retina after retinectomy (Stone, 1950; Yoshii et al., 2007; Miyake and Araki, 2014) to regeneration of specific neuron types after more localized ablation procedures (Choi et al., 2011; Powell et al., 2016; Langhe et al., 2017), which allowed the identification of several stem/progenitor cell populations. One major source of retinal regeneration are the resident MG cells in the INL. These glia are able to re-enter the cell cycle, dedifferentiate into retinal progenitors, and regenerate new retinal neurons when stimulated by either growth factors or various mechanical, neurotoxic, and light-induced injuries (Fausett and Goldman, 2006; Bernardos et al., 2007; Fimbel et al., 2007; Gallina et al., 2014; Powell et al., 2016; Langhe et al., 2017). In addition to MG cells, activation of CMZ progenitors at the retinal margin have been shown to contribute to regeneration in response to some injuries either involving peripheral retina damage or full retinal ablation (Raymond et al., 1988; Negishi et al., 1992; Stenkamp et al., 2001; Centanin et al., 2014; Miyake and Araki, 2014; Langhe et al., 2017).

Although endogenous cellular sources for retinal regeneration have been reported in all vertebrates tested so far including fish, amphibians, chicken, and mammals, the regenerative capacity is highly variable and usually strongly reduced in amniote species. For example, regeneration in birds and rodents through activation of MG (Fischer and Reh, 2001; Karl et al., 2008) or CMZ progenitors (Moshiri and Reh, 2004; Fischer, 2005; Jian et al., 2009) mostly occurs at embryonic or early postnatal stages (Fischer and Reh, 2001, 2003b; Ueki et al., 2015), and frequently requires transgenic modifications (Jorstad et al., 2017; Rueda et al., 2019), sufficiently severe injury (Fischer and Reh, 2001), and/or addition of exogenous growth factors (Fischer and Reh, 2002; Close et al., 2006; Karl et al., 2008). Furthermore, the achieved regenerative response is limited in terms of duration (Fischer and Reh, 2003b; Jian et al., 2009; Ueki et al., 2015), capacity to generate particular cell types (Fischer and Reh, 2001; Close et al., 2006; Karl et al., 2008), and ability to restore correct neural architecture (Fischer, 2005). Surprisingly, however, little is yet known about morphogenesis and regeneration of retinal tissues in the squamate group of reptiles (i.e., lizards and snakes), which occupies a key phylogenetic position within amniotes (Eernisse and Kluge, 1993) and exhibits unique regeneration features in many tissues (Kaslin et al., 2008; Chang et al., 2009; Kusumi and Fisher, 2014; Salomies et al., 2019). In the eye, a few reports have described the relatively well-conserved characteristics of retinal glia in reptiles (Dahl et al., 1985; Gaur et al., 1988; Casañas et al., 2011; Romero-Alemán et al., 2012; Todd et al., 2015), and we recently showed that snakes and lizards possess a proliferative CMZ-like peripheral retina (termed retinociliary junction or RCJ) that acts as a source of progenitors compensating for lifelong ocular growth (Eymann et al., 2019). These results suggest that, similarly to other vertebrate groups, at least a subset of retinal cells in reptiles might retain a capacity for retinal regeneration. Yet, besides assessment of optic nerve regeneration in a few lizard and snake species (Rio et al., 1989; Beazley et al., 1998; Lang et al., 2002; Dunlop et al., 2004), scarcely any studies of acute retinal damage have been developed in reptiles (Yaqub and Eldred, 1993; Ávila-Mendoza et al., 2016), and virtually nothing is known about the regenerative response following retinal injury in squamates. Hence, studying such group is absolutely necessary to fully understand the evolution and diversity of retinal regeneration mechanisms across vertebrates. Furthermore, it might offer a new framework for identifying new targets and/or developing new therapeutic approaches for regenerative medicine.

Here, we assess both the damage and regenerative responses of the squamate retina exposed to excitotoxic neuronal injury, using the bearded dragon lizard (*Pogona vitticeps*) as a main model because of its characterized embryonic and adult retinal features (Hiscock and Straznicky, 1991; Straznicky and Hiscock, 1994; Eymann et al., 2019). Furthermore, the bearded dragon is currently emerging as a new model organism for craniofacial biology and development (Ollonen et al., 2018; Salomies et al., 2019), and our recent identification of a source of highly proliferating progenitors persisting postnatally in this lizard (Eymann et al., 2019) makes it a likely candidate to possess regenerative neurogenic capacity in response to retinal injury. Our findings reveal for the first time that squamates such as bearded dragons hold the potential for postnatal retinal regeneration following acute injury. Whereas progenitor activity at the retinal peripheral margin is not increased in response to neurotoxin-induced damage, proliferating cells identified as MG in central retina re-enter the cell cycle, de-differentiate to acquire a progenitor-like phenotype, and re-express proneural markers. Importantly, the overall MG responses appear relatively robust in bearded dragon when compared to absent or neonatal-limited regeneration reported in other amniotes. Altogether, this set of new results helps to complete our evolutionary understanding of regenerative potential of the vertebrate retina, and further highlights the major importance of glial cells in retinal regeneration. Furthermore, our work offers a new powerful animal model to elucidate the developmental and evolutionary bases of retinal regeneration within amniotes.

## Materials and Methods

### Animals

Bearded dragon lizards (*Pogona vitticeps*) were obtained from our animal facility at the University of Helsinki or from private breeders. All specimens analyzed were at juvenile stage (2–6 months after hatchling). All reptile captive breedings and experimental protocols were approved by the Laboratory Animal Centre (LAC) of the University of Helsinki and/or the National Animal Experiment Board (ELLA) in Finland (license numbers ESLH-2007-07445/ym-23, ESAVI/7484/04.10.07/2016, and ESAVI/13139/04.10.05/2017).

### Retinal Damage and Pulse-Chase Experiments

Prior to treatment, animals were habituated to handling and hand/syringe feeding for at least 7 days. To induce retinal damage, animals were first anesthetized with intravenous injection of propofol (5 mg/kg body weight) in the ventral coccygeal vein. A Hamilton syringe with a 26-gauge needle was used for intraocular injection of neurotoxins [1 or 2 μmol *N*-methyl-D-aspartate (NMDA)] and vehicle/saline solution (control) in the vitreous chamber of left and right eyes, respectively, as previously described in chicken (Fischer et al., 1998). Injections were made through the dorsal eye lid at the equatorial plane of the eye. Following injections, animals were placed in a warm, dry recovery terrarium to prevent hypothermia. Upon full recovery from anesthesia (approximately 3 h post-treatment), animals received first feeding with 5-bromo-2′-deoxyuridine (BrdU) solution by squirting the solution into the mouth, as described before (Eymann et al., 2019; Salomies et al., 2019). BrdU was subsequently administered twice daily (80 mg/kg body weight) for a period of 3 days. In double pulse-chase experiments, 5-ethynyl-2′-deoxyuridine (EdU) solution (80 mg/kg body weight) was administered analogous to BrdU for a period of 3 days twice daily. An average number of 3 animals per time points (3, 6, 9, 14, 18, and 24 days post-injection;18 animals in total) were allowed to recover from injection prior to euthanasia. To minimize potential differences in food intake linked to impaired vision, all animals were continued to be hand fed over the entire course of the experiments.

### Immunohistochemistry (IHC) and Apoptosis Assays on Paraffin Sections

Treated and control eyes were first dissected and inspected for any potential gross damage incurred by the injection procedure. Eyes were punctured nasally to facilitate solvent penetration during subsequent histological processing. Samples were first fixed overnight in 4% paraformaldehyde (PFA) at 4°C, dehydrated in a graded alcohol series, embedded in paraffin, and finally sectioned coronally at 7 μm. IHC fluorescent staining was performed as described previously (Salomies et al., 2019). In short, antigens were unmasked by heat-induced epitope retrieval (HIER) and detected using overnight incubation at 4°C with primary antibodies known to recognize reptile and/or chicken epitopes: 5-bromo-2′-deoxyuridine (BrdU; 1:300, rat monoclonal, Abcam, cat# ab6326, RRID:AB_305426), doublecortin (DCX; 1:200, rabbit polyclonal, Cell Signaling Technology, cat# 4604, RRID: AB_561007), glial fibrillary acidic protein (GFAP; 1:300, mouse monoclonal, LifeSpan, cat# LS-C88015-100, RRID: AB_1795608), proliferating cell nuclear antigen (PCNA; 1:200, mouse monoclonal, BioLegend, cat# 307901, RRID: AB_314691), neuronal-specific RNA-binding proteins HuC/D (HuC/D; 1:200, mouse monoclonal, Thermo Fisher Scientific, cat# A-21271, RRID: AB_221448), and SRY-box 9 (SOX9; 1:400, rabbit polyclonal, Millipore, cat# AB5535, RRID: AB_2239761). Last, sections were incubated for 1 h at room temperature with Alexa Fluor-conjugated secondary antibodies (Alexa Fluor-488: 1:500, goat anti-rabbit IgG, Thermo Fisher Scientific, cat# A-11008, RRID: AB_143165; Alexa Fluor-568: 1:500, goat anti-rabbit IgG, Thermo Fisher Scientific, cat# A-11011, RRID: AB_143157). Cell death was detected using terminal deoxynucleotidyl transferase dUTP nick end labeling (TUNEL) method (*in situ* cell death detection kit, Roche), as previously described in reptiles including bearded dragon (Milinkovitch et al., 2013; Salomies et al., 2019). Briefly, rehydrated paraffin sections were washed in phosphate buffered saline (PBS) before addition of 50 μl TUNEL staining solution per section, and slides were incubated in a dark, humidified chamber at 37°C for 1 h. Following both IHC and TUNEL stainings, slides were mounted with Fluoroshield mounting medium (Sigma-Aldrich) containing 4′,6′-diamidino-2-phenylindole (DAPI).

### Double *in situ* Hybridization (ISH)-IHC Labeling

Double ISH-IHC labeling was performed on paraffin sections as described previously (Eymann et al., 2019). Briefly, rehydrated sections were first pre-treated with proteinase K (Roche); then acetylated with 0.25% acetic anhydride in 0.1M triethanolamine buffer; and re-fixed with 4% PFA. Finally, sections were hybridized overnight at 60–65°C with digoxigenin (DIG)-labeled antisense riboprobes corresponding to *Pogona vitticeps* paired box 6 (*Pax6*, 654 bp) and apolipoprotein E (*ApoE*, 817 bp). Corresponding sense riboprobes were used as negative controls. After hybridization, sections were washed and incubated with alkaline phosphatase-conjugated anti-DIG antibodies (1:2500, sheep polyclonal, Sigma-Aldrich, cat# 11093274910, RRID:AB_2734716). For colorimetric visualization of hybridization, sections were stained with a solution containing 5-bromo-4-chloro-3-indolyl phosphate and nitro blue tetrazolium. Finally, slides were washed in PBS solution and processed for IHC without HIER step.

### Imaging and Quantification

Bright field images were acquired with an AX70 microscope equipped with a DP70 camera (Olympus). Fluorescent staining was imaged using a Nikon Eclipse 90i fluorescence microscope equipped with a Hamamatsu Flash4.0 camera. Bright field and fluorescent images were overlaid and/or processed with Adobe Photoshop CC (RRID:SCR_014199) using photomerge tool without blending option for tiled image merging and linear levels adjustment for visualization improvement. Measurement of individual retinal layer thickness and counting of fluorescent-positive cells in acquired images were performed using Fiji/ImageJ software (RRID:SCR_002285; Schindelin et al., 2012). Morphological features of the retina detectable by DAPI staining were used to identify the retinociliary junction (RCJ) as well as the central and peripheral retina regions, as previously described (Eymann et al., 2019). The RCJ was defined as the pseudostratified region at the extreme periphery of the retina between the monolayered ciliary epithelium and the stratified peripheral retina (Supplementary Figures S1B,C; Eymann et al., 2019). The retinal periphery was defined as a 200 μm wide region of stratified (i.e., observable OPL and IPL) retinal periphery immediately adjacent to the RCJ. The central retina area used for quantification covered a region of at least 400 μm wide in the central retina located at least 300 μm dorsal from the ONH and outside the foveal pit (Supplementary Figures S1B,C). For each experimental stage and/or treatment, TUNEL, PCNA, and/or BrdU quantifications were acquired from at least 3 (for central and peripheral retinas) or 4 (for RCJ) representative retinal areas sectioned in coronal plane per animal eye, using an average number of 3 treated or control eyes per stage. Quantification of apoptotic and/or proliferative cells in individual layers of central and peripheral retinas was normalized to the surface area because of the absence of cell nuclei in some layers under normal conditions. As previously described (Eymann et al., 2019), the size and nuclei number of the RCJ are highly variable among individuals in contrast to the overall range of proliferating cell number. Therefore, proliferative activity in the RCJ was quantified as before (Eymann et al., 2019) based on the absolute rather than relative number of PCNA-immunoreactive cell nuclei per RCJ. Statistical significance between saline control and/or treated eyes was determined by one-way ANOVA followed by *post hoc* pairwise Student’s *t*-test in Microsoft Excel software (RRID:SCR_016137).

## Results

### Neurotoxic-Induced Damage of Squamate Retinal Tissues

**FIGURE 1:**
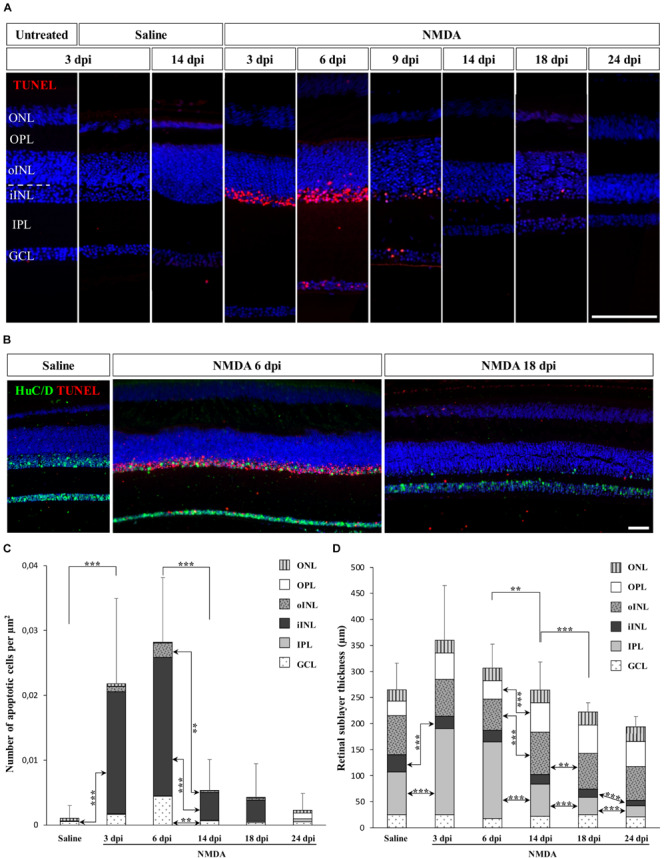
NMDA-induced damage in the bearded dragon central retina. **(A)** TUNEL apoptotic assay (red staining) in the central retina of untreated, saline-treated (saline), or NMDA-treated (NMDA) eyes at 3, 6, 9, 14, 18, and 24 days post-injection (dpi). Because of the constant, low number of apoptotic cells detected in both untreated and saline-treated retinas at all time points, only controls at 3 dpi are shown later in the manuscript. Cell nuclei are counterstained with DAPI (blue). The different retinal layers are indicated (ONL, outer nuclear layer; OPL, outer plexiform layer; INL, inner nuclear layer; oINL, outer INL; iINL, inner INL; IPL, inner plexiform layer; GCL, ganglion cell layer), and the dashed white line outlines the transition between oINL and iINL layers. Scale bar, 100 μm. **(B)** HuC/D immunohistochemistry (IHC, green staining) combined with TUNEL apoptotic assay (red staining) showing the distribution of apoptotic cells in iINL and GCL layers at 3 dpi (saline) or 6 and 18 dpi (NMDA). Scale bar, 100 μm. **(C,D)** Quantification of the number of apoptotic cells per surface area identified by TUNEL apoptotic assay **(C)** or thickness measurement **(D)** in all indicated morphologically distinct retinal layers (see fill pattern code in top right corners) from saline-treated (saline) or NMDA-treated (NMDA) eyes at 3, 6, 14, 18, and 24 dpi. For clarity of the graphs, only highly significant values based on Student’s *t*-test for both the whole-retina (top asterisks) and individual layers (asterisks between columns) are indicated (**p-value <0.01; ***p-value <0.001).

Many studies have focused on the unique ability of “lower” vertebrates such as fish and amphibians to regenerate a substantial fraction of the neural retina, but little is known about the response to retinal damage in squamates, which are more closely related to mammals. To assess whether retinal neurons could be selectively destroyed and regenerated in the bearded dragon model, we used *N*-methyl-D-aspartate (NMDA)-mediated retinal damage, one of the most commonly used retinal degeneration models in animals (Niwa et al., 2016). Furthermore, although NMDA-induced retinal excitotoxicity has not yet been explored in squamates, this treatment has a well-established procedure (see, e.g., Fischer et al., 1998) and studies in all other tested vertebrate groups have shown that intravitreal injection of NMDA leads to the selective ablation of bipolar and amacrine cells of inner INL (iINL) and GCL retinal layers (Fischer et al., 1998; Powell et al., 2016), thus allowing direct comparison of the regeneration processes among various vertebrate species. Therefore, we initially examined the location and extent of retinal damage induced by similar intraocular injection of NMDA, by comparing apoptotic DNA fragmentation patterns and histological changes of retinal tissues sampled at different days post-injection (dpi). In control eyes treated with saline solution, little to no apoptotic cells were detected in the whole-retina at any time following injection, and all retinal layers were morphologically indistinguishable from untreated eyes, indicating that the mechanical damage following injection procedure does not trigger severe retinal injury (Figure 1A). In contrast, eyes treated with NMDA show widespread apoptosis in the central retina starting from 3 dpi. In particular, a high proportion of amacrine cells from GCL and iINL are sensitive to NMDA, as revealed by the decrease of both cell density and immunohistochemical (IHC) detection of amacrine cell marker such as neuronal-specific RNA-binding proteins HuC/D (Fausett and Goldman, 2006; Rueda et al., 2019) in these two layers (Figure 1B). Importantly, although different levels of NMDA resulted in similar location of apoptotic cells in bearded dragon, we found that damage elicited by a relatively large dose (2 μmol) was more efficient and consistent across the whole-retina (Figures 1A,B and Supplementary Figures S1B,C) while keeping the overall retinal architecture intact, so this amount was used in subsequent experiments. This is reminiscent of reports in chicken showing that the same NMDA dose is required to induce major, specific retinal damage and response (Fischer and Reh, 2001). In our bearded dragon model, the number of apoptotic cells is maximal between 3 and 6 dpi in both GCL and iINL layers, as revealed by detection of DNA fragmentation, before significantly decreasing at later time points until 24 dpi (Figures 1A,C). In line with this ongoing apoptotic process, our data further indicate significant changes in the thickness of some retinal layers over the course of NMDA treatment (Figures 1A,D). Dramatic changes especially occur in the IPL, which shows first a large increase in thickness at 3 and 6 dpi followed by a large, progressive thinning eventually reaching a significantly hypotrophied state at 18 and 24 dpi (Figures 1A,D). In addition to the IPL, the thickness of the iINL is significantly affected in NMDA-treated retinas over the course of the treatment (Figure 1D). Amacrine cells are well-known to be the major iINL cell type in vertebrate retinas, so the observed reduced thickness of this layer is coherent with a loss of amacrine cells induced by NMDA treatment (Figure 1B). Interestingly, however, no significant differences were observed in the thickness of the GCL, which only contains some displaced amacrine cells among other cell types. Altogether, these results indicate that intravitreal injection of NMDA in bearded dragons specifically affects GCL and iINL regions of central retina, with apoptotic and morphological changes being comparable to descriptions in other vertebrates. However, when compared to equivalent NMDA-mediated neurotoxicity in chicken or fish (Fischer et al., 1998; Powell et al., 2016), both increased apoptosis and changes in retinal layer thickness appear several days later post-treatment and last for a longer period, suggesting that the overall response is prolonged in our lizard model.

### NMDA-Induced Response at the Peripheral Retina

**FIGURE 2:**
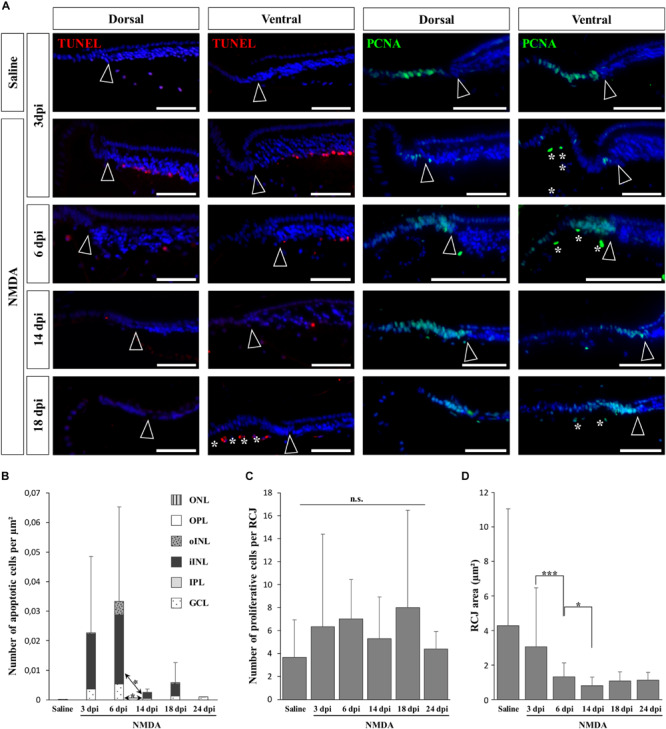
NMDA-induced response at the retinal margin. **(A)** TUNEL apoptotic assay (left panels, red staining) or IHC detection of PCNA proliferation marker (right panels, green staining) in the peripheral retina of saline-treated (saline) or NMDA-treated (NMDA) eyes at 3, 6, 14, and 18 dpi. Cell nuclei are counterstained with DAPI (blue). Open arrowheads delimitate the most peripheral edge of the stratified peripheral retina, and white asterisks indicate the presence of TUNEL- and/or PCNA-positive cellular debris inside the vitreous body. Scale bars, 100 μm. **(B)** Quantification of the number of apoptotic cells per surface area, as identified by TUNEL apoptotic assay, in all indicated morphologically distinct layers of peripheral retina (ONL, outer nuclear layer; OPL, outer plexiform layer; INL, inner nuclear layer; oINL, outer INL; iINL, inner INL; IPL, inner plexiform layer; GCL, ganglion cell layer) from saline-treated (saline) or NMDA-treated (NMDA) eyes at 3, 6, 14, 18, and 24 dpi (see fill pattern code in top right corner). **(C,D)** Quantification of the number of proliferative cells identified by PCNA IHC **(C)** or area measurement **(D)** in the pseudostratified region (retinociliary junction, RCJ) between the monolayered ciliary epithelium and the stratified peripheral retina from saline-treated (saline) or NMDA-treated (NMDA) eyes at 3, 6, 14, 18, and 24 dpi. Student’s *t*-test was used for statistical analysis (*p-value <0.05; ***p-value <0.001; n.s., non-significant).

In vertebrates such as amphibians and fish, endogenous progenitors residing in the CMZ at the retinal peripheral margin have already been shown to contribute to lifelong retinal growth but also to regeneration following various retinal injuries (see, e.g., Reh and Levine, 1998; Hitchcock et al., 2004). Since a similar peripheral CMZ-like region, the RCJ, exists in bearded dragon lizards (Eymann et al., 2019), we first tested whether these progenitors could proliferate and regenerate neurons in response to NMDA-induced retinal damage. We particularly focused our analysis on the peripheral retina region next to the RCJ region, as studies in other vertebrates have shown that essentially this retinal region could regenerate using CMZ precursors (Centanin et al., 2014; Miyake and Araki, 2014), and long-distance migration of progenitor cells within the retina has not been previously observed. In our lizard model, although the RCJ itself was devoid of apoptotic cells at all time points following NMDA injection, we first confirmed the presence of an apoptotic wave in the adjacent retinal periphery (Figure 2A, left panels). Similarly to the injured central retina, the number of peripheral apoptotic cells peaks at 3 and 6 dpi in both GCL and iINL layers (Figures 2A,B). At these two stages, the apoptotic cells form a continuous layer expanding from the central retina to the edge of the laminated neural retina (Supplementary Figures S1B,C), before decreasing in number to reach basal levels already during the second week post-injection (Figures 2A,B). It is worth noting that some disparity in the number of iINL apoptotic cells exists between the dorsal and ventral regions of the retinal periphery, thus explaining the observed variation in our quantification (Figure 2B and Supplementary Figures S1B,C). Such dorso-ventral fluctuation might reflect differences in tissue development and/or structure (Eymann et al., 2019) or heterogeneous treatment effect (Vihtelic et al., 2006). Strikingly, although IHC detection of proliferating cell nuclear antigen (PCNA) proliferation marker confirms the presence of proliferating cells at the RCJ at all examined time points (Figure 2A, right panels), no signs of increased proliferation were observed over the course of NMDA treatment when compared to undamaged saline controls (Figures 2A,C). Similarly, other parameters that were previously linked to CMZ-mediated regeneration in fish and amphibians (Centanin et al., 2014; Miyake and Araki, 2014) but also to increased progenitor activity at the RCJ in this lizard (Eymann et al., 2019), including morphological expansion and increased thickness of the retinal margin as well as changes in stem/progenitor marker expression, were not detected (Figures 2A,D and data not shown). On the contrary, our quantification of the RCJ area rather indicates a significant shrinkage in NMDA-treated retinas (Figure 2D). Altogether, these results indicate that in contrast to the CMZ of amphibians and fish, progenitors at the RCJ are unlikely to strongly contribute to retinal regeneration beyond their physiological activity, as they do not respond to NMDA treatment.

### NMDA-Induced Proliferative Response at the Central Retina

**FIGURE 3:**
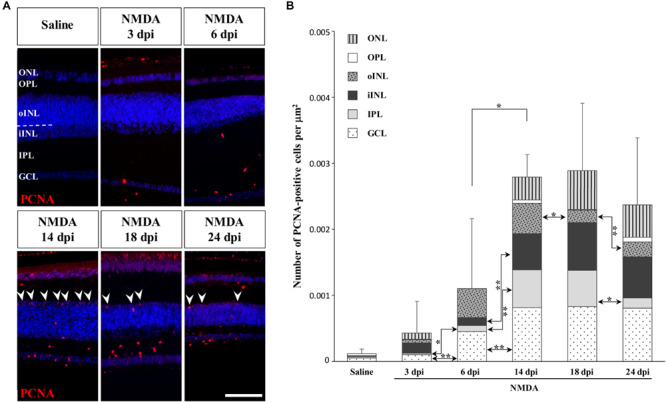
NMDA-induced proliferative response at the central retina. **(A)** IHC detection of PCNA proliferation marker (red staining) in the central retina of saline-treated (saline) or NMDA-treated (NMDA) eyes at 3, 6, 14, 18, and 24 dpi. Cell nuclei are counterstained with DAPI (blue). The different retinal layers are indicated (ONL, outer nuclear layer; OPL, outer plexiform layer; INL, inner nuclear layer; oINL, outer INL; iINL, inner INL; IPL, inner plexiform layer; GCL, ganglion cell layer), and the dashed white line marks the transition between oINL and iINL layers. Arrowheads indicate PCNA-positive cells at the outer edge of oINL. Scale bar, 100 μm. **(B)** Quantification of the number of PCNA-positive proliferative cells per surface area, as identified by PCNA IHC, in all indicated morphologically distinct retinal layers (see fill pattern code in top left corner) from saline-treated (saline) or NMDA-treated (NMDA) eyes at 3, 6, 14, 18, and 24 dpi. Significant values based on Student’s *t*-test for both the whole-retina (top asterisks) and individual layers (asterisks between columns) are indicated (*p-value <0.05; **p-value <0.01).

**FIGURE 4:**
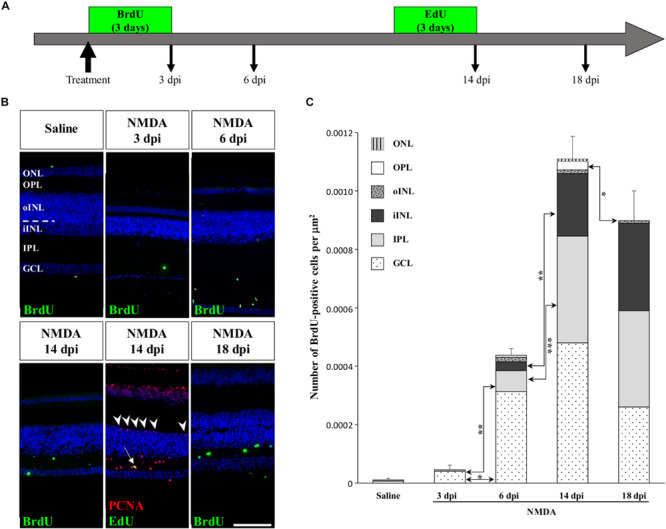
Pulse-chase assays to assess proliferation at the central retina in response to NMDA treatment. **(A)** Scheme of experimental strategies showing BrdU or EdU pulse-chase time points in saline control and NMDA-treated juvenile animals. Retinal tissues were collected at 3, 6, 14, and 18 dpi. **(B)** IHC detection of different proliferation markers, including BrdU alone (green) or EdU (green) combined with PCNA (red), in the central retina of saline-treated (saline) or NMDA-treated (NMDA) eyes at indicated days post-injection (dpi). Cell nuclei are counterstained with DAPI (blue). The different retinal layers are indicated (ONL, outer nuclear layer; OPL, outer plexiform layer; INL, inner nuclear layer; oINL, outer INL; iINL, inner INL; IPL, inner plexiform layer; GCL, ganglion cell layer), and the dashed white line marks the transition between oINL and iINL layers. The white arrow indicates a rare EdU/PCNA double-positive cell in the IPL. Arrowheads indicate PCNA-positive cells at the outer edge of oINL. Scale bar, 100 μm. **(C)** Quantification of the number of BrdU-positive proliferative cells per surface area, as identified by BrdU IHC, in all indicated morphologically distinct retinal layers (see fill pattern code in top left corner) from saline-treated (saline) or NMDA-treated (NMDA) eyes at 3, 6, 14, and 18 dpi. Significant values based on Student’s *t*-test for individual retinal layers are indicated (*p-value <0.05; **p-value <0.01; ***p-value <0.001).

To determine if other cellular sources could serve as retinal progenitors in response to acute damage, we next characterized the proliferation pattern of different sublayers of central retina following NMDA treatment (Figures 3, 4). Whereas saline controls exhibit very few proliferating cells at all examined time points, injured individuals show drastic changes in retinal proliferation over the course of NMDA treatment, with PCNA-positive cells distributed among different layers (Figure 3A). Starting from 3 dpi, the number of proliferating cells first increases significantly in inner retinal layers such as GCL and IPL, reaching maximal levels at 14 dpi. At this latter stage, PCNA-positive cells are relatively increased in additional retinal layers including INL and ONL, and the whole-retina achieves maximal proliferation (Figures 3A,B). Of note is the rise of cell proliferation at 14 dpi on the outer edge of the INL, forming a continuous sublayer of PCNA-positive cells (Figure 3A, arrowheads). Importantly, this area is the first retinal region displaying a significant decrease in the number of proliferative cells already from 18 dpi, indicating a more transient proliferative behavior of oINL cells (Figures 3A,B). In contrast, the proliferative activity of the IPL, one of the first active regions, is significantly reduced later at 24 dpi (Figure 3B). To confirm the observed proliferation patterns but also track the progeny of early proliferating cells in NMDA-damaged central retina, we next labeled mitotically active cells with 5-bromo-2′-deoxyuridine (BrdU) for 3 days following NMDA treatment (Figure 4A). Similarly to PCNA stainings, IHC detection of BrdU incorporation in central retina indicates an initial rapid increase of BrdU-positive cells in the innermost retinal layers (GCL, IPL, and iINL) over the first 2 weeks post-treatment (3–14 dpi), and then a subsequent reduction at later stages (18 dpi; Figures 4B,C). Interestingly, although these observations parallel the results of PCNA immunodetection in the abovementioned retinal layers, direct comparisons of both total and layer-specific amount of labeled cells indicate drastically lower numbers of BrdU-positive cells, when compared to PCNA (Figures 3A,B). Furthermore, our BrdU experiments failed to detect positive cells in the PCNA-positive sublayer at the outer edge of the INL, even when initiating BrdU incorporation at different time points post-treatment or by using alternative dyes such as 5-ethynyl-2′-deoxyuridine (EdU) to identify cell proliferation (Figure 4B and data not shown). As a result, proliferating cells remain nearly exclusively in the innermost retinal layers, with less than 25% of BrdU-positive cells appearing in the outer retinal portion (Figure 4C). The observed discrepancies between PCNA expression and BrdU/EdU incorporation immediately after the respective labeling pulse might derive from the different specificity of these markers toward cell cycle phases, BrdU/EdU being only specific to S phase. However, the overall lower number of BrdU- and EdU-positive cells in central retina throughout the experiment rather suggest a low incorporation of the dyes into dividing retinal cells. Based on our previous study where 7 days were required to efficiently label retinal progenitors with BrdU in this lizard model (Eymann et al., 2019), we suspect that this deficiency could be linked to the relatively short period of dye exposure, which is restricted here by the timing of damage response. As also evident from the RCJ shrinkage observed at the peripheral region, other contributing factors could include metabolic changes in NMDA-injured animals, reducing BrdU/EdU absorption in lizards already known to exhibit a low metabolic rate. However, despite the reduced BrdU/EdU labeling, the discrete laminar localization and sequential initiation of proliferation indicate the activation of distinct populations of proliferating cells in response to NMDA treatment.

### Early Activation of Microglia/Macrophages in Response to Injury

**FIGURE 5:**
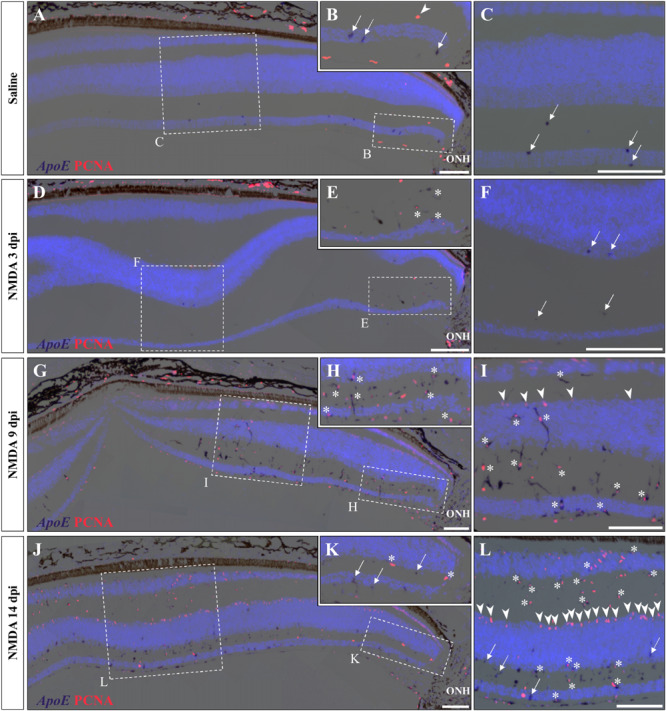
Microglial response to NMDA treatment. **(A–L)** Double ISH/IHC staining of microglia/macrophage marker *ApoE* (purple) with proliferation markers PCNA (red) in the central retina of bearded dragon treated with saline **(A–C)** or NMDA at 3 dpi **(D–F)**, 9 dpi **(G–I)**, and 14 dpi **(J–L)**. Retinal layers are highlighted by DAPI (light blue) nuclear counterstaining. Insets show high magnifications of positive staining in inner retinal layers near the optic nerve head (ONH) **(B,E,H,K)** or in central retina layers **(C,F,I,L)**. Arrows indicate *ApoE*-positive, PCNA-negative cells **(B,C,F,K,L)**; arrowheads indicate *ApoE*-negative, PCNA-positive cells at the outer edge of oINL **(B,I,L)**; asterisks indicate double *ApoE*/PCNA-positive cells **(E,H,I,K,L)**. Scale bars, 100 μm.

A variety of acute retinal injuries in different vertebrate species have been shown to induce the early recruitment of proliferative phagocytic glial cells (microglia) and/or macrophages in inner retinal layers such as GCL and IPL, a process associated with the clearance of cellular debris (Maier and Wolburg, 1979; Fischer et al., 1998, 2014; Vihtelic and Hyde, 2000; Fischer and Reh, 2001; Lillo et al., 2001; Yurco and Cameron, 2005; Fimbel et al., 2007). Interestingly, in these two layers a progressive increase of both PCNA- and BrdU-positive cells was observed between 3 and 14 dpi (Figures 3A,B, 4B,C), so we proceeded to investigate whether similar presumptive microglia/macrophage cell types are also recruited upon NMDA injury in our lizard model. To molecularly characterize the identity of the early proliferating population, we used double IHC-*in situ* hybridization (ISH) stainings against proliferation (PCNA or BrdU) and microglia/macrophage(apolipoprotein E (*ApoE*); Anderson et al., 2019) markers. In saline-treated control retinas, a few non-proliferative, *ApoE*-positive resident microglia/macrophage are scattered in the GCL but rarely observed in the IPL (Figures 5A–C; white arrows). Upon NMDA treatment at 3 dpi, a cluster of *ApoE*-positive cells appears near the optic nerve head (ONH) in the IPL, GCL, and nerve fiber layer (Figures 5D–F), as would be expected from infiltrating microglia/macrophages invading the retina from the ONH region (Maier and Wolburg, 1979; Fischer et al., 1998; Conedera et al., 2019). Furthermore, in contrast to the resident cells observed in control retinas, these presumptive reactive microglia/macrophages exhibit a distinct amoeboid morphology and express proliferation markers (Figures 5E,F; white asterisks). At 9 dpi, these reactive cells dramatically increase in number and spread to more peripheral locations, but mainly remain confined to the innermost half of the retina (Figures 5G–I). Interestingly, however, a few PCNA/*ApoE* double-positive cells exhibiting radially elongated shapes start to appear in both IPL and INL regions at 9 dpi (Figures 5H,I; white asterisks), suggesting a movement of reactive microglia/macrophages toward more apical retinal layers. At 14 dpi, *ApoE*-expressing cells are scattered throughout most of the retinal layers, from central to peripheral retina (Figures 5J–L), but only few of these cells remain proliferative in the innermost portion of the retina (Figures 5K,L; white arrows). Importantly, however, the *ApoE* marker was never detected in the second transient population of proliferating cells at the outer edge of the INL (Figures 5I,L; white arrowheads), further indicating that these cells are not part of the initial reactive microglia/macrophage response.

### Activation of Müller Glia Cells in Response to Injury

**FIGURE 6:**
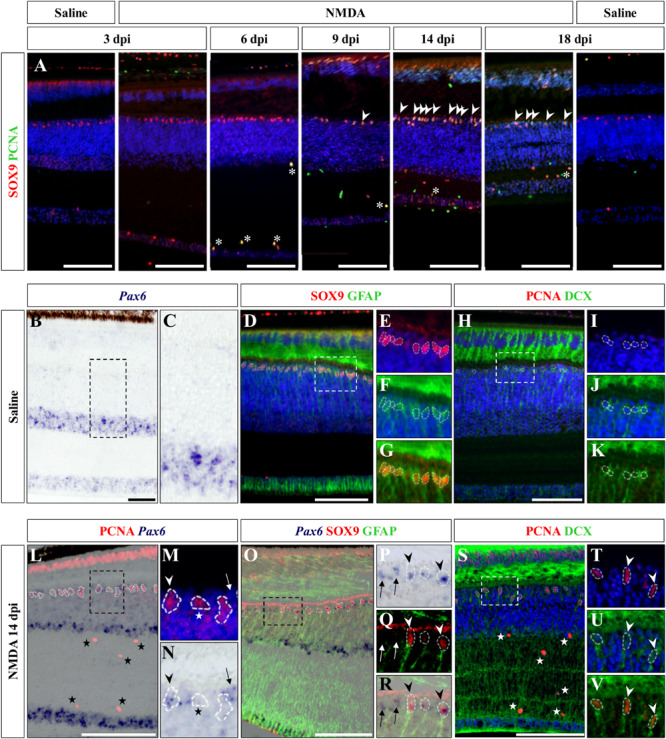
Characterization of Müller glia (MG) response at the central retina. **(A)** Double IHC staining of proliferation marker PCNA (green) and MG marker SOX9 (red) in the central retina of saline-treated (saline) or NMDA-treated (NMDA) eyes at 3, 6, 9, 14, and 18 dpi. White asterisks and arrowheads indicate SOX9/PCNA double-positive putative microglia or MG, respectively. **(B–K)** ISH detection of progenitor marker *Pax6* (purple) **(B,C)**, double IHC staining of MG markers SOX9 (red) and GFAP (green) **(D–G)**, or double IHC staining of proliferation marker PCNA (red) and proneural marker DCX (green) **(H–K)** in the central retina of saline-treated bearded dragon at 3 dpi. Retinal layers are highlighted by DAPI (blue) nuclear counterstaining in panels **(D–F,H–J)**. Insets show high magnifications of INL retinal layers for individual **(E,F,I,J)** or combined **(G,K)** fluorescent channels. **(L–V)** ISH detection of *Pax6* (purple) combined with PCNA IHC (red) **(L–N)** or double SOX9 (red) and GFAP (green) IHC **(O–R)**, or double IHC staining of PCNA (red) and DCX (green) **(S–V)** in the central retina of NMDA-treated bearded dragon at 14 dpi. Insets show high magnifications of PCNA alone with DAPI **(M,T)**, *Pax6* **(N,P)**, SOX9/GFAP **(Q)**, *Pax6*/SOX9/GFAP **(R)** DCX with DAPI **(U),** and PCNA/DCX **(V)**. Arrowheads indicate putative MG or MG-derived progenitor cells **(M,N,P–R,T–V)**. Stars and arrows mark single PCNA- **(L–N,S)** or *Pax6*-positive **(M,N,P–R)** cells, respectively.

We next aimed at assessing the identity of the cells proliferating in the outer INL edge at 14 dpi following retinal damage. Besides their location, our observations so far show that these cells exhibit elongated nuclei and virtually lack *ApoE* expression, suggesting that they might correspond to reactive MG cells. Indeed, retinal MG are well-known to adopt stem/progenitor characteristics in injured retinas of other vertebrates (Fausett and Goldman, 2006; Bernardos et al., 2007; Fimbel et al., 2007; Gallina et al., 2014; Powell et al., 2016; Langhe et al., 2017), and despite the manifold variations in experimental setups and outcomes, there are shared commonalities in the regenerative reaction of these cells to damage: cell cycle re-entry, dedifferentiation into progenitors, which may undergo interkinetic nuclear migration (INM), and ultimately differentiation into new neurons that integrate into the appropriate retinal structures. To first confirm the identity of the proliferative oINL cell population in our model, we labeled control and injured retinal tissues at all experimental time points for PCNA together with SRY-box 9 (SOX9), a conserved marker of reptile MG cells (Todd et al., 2015). While a distinct sublayer of oINL cells show strong SOX9 expression in both control and treated retinas, as previously described for lizards (Romero-Alemán et al., 2012; Todd et al., 2015), proliferating PCNA/SOX9 double-positive cells in this layer only appear in injured retinas starting from 9 dpi (Figure 6A; white arrowheads). In line with our quantification of cell proliferation (see Figure 3B), the amount of PCNA/SOX9 cells peaks at 14 dpi, with most of SOX9-positive cells at the outer edge of oINL co-expressing PCNA, before reducing at 18 dpi (Figure 6A). This double labeling experiment already suggests that a significant proportion of PCNA-positive cells from oINL are in fact proliferating MG cells. In other tested vertebrate species, reactive MG usually dedifferentiate into retinal progenitors following re-entry in the cell cycle, as marked by the upregulation of retinal progenitor markers such as paired box 6 (*Pax6*) in fish (Bernardos et al., 2007; Thummel et al., 2008), amphibians (Langhe et al., 2017), birds (Fischer and Reh, 2001), and mammals (Karl et al., 2008; Joly et al., 2011; Jorstad et al., 2017). Therefore, we further characterized the PCNA-expressing presumptive MG population, by assessing the expression pattern of *Pax6*. In control retinas of bearded dragon, *Pax6* is expressed in non-proliferative amacrine and ganglion cells of both GCL and inner half of INL (Figures 6B,C), as previously described in other lizard species (Rodger et al., 2006; Todd et al., 2015). However, in addition to the abovementioned positive layers, NMDA-injured retinas at 14 dpi exhibit another band of *Pax6* expression co-localizing with PCNA-expressing presumptive MG cells at the oINL (Figures 6L–N; arrowheads), suggesting that these cells correspond to MG-derived progenitor cells (MGDPs). In support of that, triple labeling using ISH against *Pax6* and IHC against two MG markers, SOX9 and glial fibrillary acidic protein (GFAP), confirms that at least a subset of cells in the outer INL edge expresses all three markers in treated tissues only (Figures 6B–G,O–R; arrowheads). Based on previous molecular characterization of retinal cell types in lizards (Dahl et al., 1985; Todd et al., 2015), the triple-labeled cells are not expected to correspond to astrocytes or inner retinal glia-like cells. In addition, some of the proliferating progenitor cells also express doublecortin (DCX), an early marker of neuronal differentiation, again pointing to a dedifferentiated MGDP state of cells in this layer (Figures 6H–K,S–V; arrowheads). Importantly, *Pax6* labeling is also observed in non-proliferative cells as well as in SOX9-negative cells at the oINL edge (Figures 6N,P,R; arrows), and DCX-positive cells also locates more centrally within the INL (Figures 6S,U,V; arrowheads), further highlighting the heterogeneity of MG dedifferentiation status but also indicating some migration of MGDPs within the INL. In contrast, although SOX9 is also expressed in proliferating microglia/macrophages within IPL and GCL (Figure 6A, asterisks), neither *Pax6* nor DCX are detected among this cell population over the whole period (Figures 6L,S; stars), making it unlikely to be migrating progeny of MGDPs. Unfortunately, however, the weak BrdU incorporation following retinal damage in our lizard model precluded any precise assessment and tracking of the fate and migration of these mitotically active cells from the oINL.

Altogether, these results indicate that reactive MG cells from the oINL have the capacity to become progenitors in the bearded dragon retina when stimulated by NMDA-induced damage. Furthermore, the contribution of MG cells to retinal regeneration as well as the heterogeneity of the MG cell population under both physiological and injured conditions has already been described in other vertebrate groups (Fischer and Reh, 2002; Jadhav et al., 2009; Ghai et al., 2010; Fischer et al., 2014).

## Discussion

**FIGURE 7:**
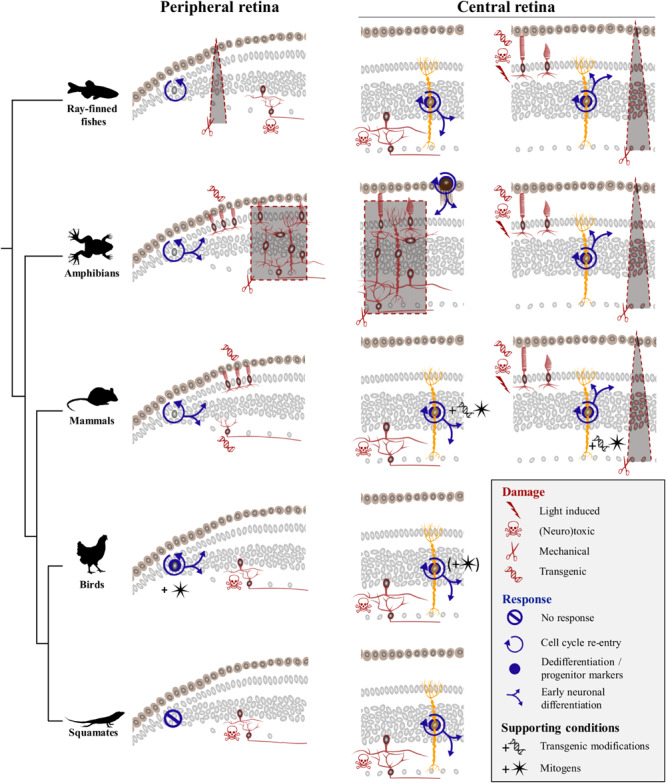
Comparison of regenerative response to various damage paradigms in different vertebrate classes. Targeted damage paradigms are indicated for both peripheral retinal margin (left) and central retina (right) with red symbols representing either light-induced (thunderbolt), neurotoxic (skull and crossbones), mechanical (scissors), or transgenic (DNA helix) damage. The location of cells affected by each treatment is shown by red colored silhouettes in peripheral and/or central retina regions. Retinal areas highlighted by red dashed lines and gray background delineate unspecific mechanical ablation of cells using locally restricted procedures (triangular shape) or full retinectomy (rectangular shape). The cellular source of the regenerative response is depicted as gray nuclei in the peripheral retinal margin or yellow-colored silhouettes corresponding to MG in the central retina. Blue icons indicate different aspects of the regenerative process, including cell cycle re-entry (circular arrow), dedifferentiation and/or expression of progenitor markers (blue disk), early neuronal differentiation (blue branching arrows), or no response (crossed out circle). Across vertebrate species, MG-mediated regeneration at central retina is widespread, with fish and amphibians being capable of restoring any retinal cell type spontaneously, and birds and mammals requiring addition of exogenous growth factors (black star symbol) or transgenic modifications (black DNA helix symbol) to induce similar response. Squamates show postnatal MG activation in response to neurotoxic treatment in the absence of additional stimulation, indicating that they lie between anamniote and other amniote groups in their regenerative capacities.

Previous vertebrate studies clearly indicate that although the retina exhibits extraordinary self-repair properties in anamniote species, retinal regeneration is more limited in mammals and birds. Furthermore, diverse cellular sources for retinal regeneration have been characterized in vertebrate species, using a variety of retinal injury and/or transgenic models. However, besides a few assessments of optic nerve regeneration after optic nerve transection or crush in lizards (Rio et al., 1989; Beazley et al., 1998; Lang et al., 2002; Dunlop et al., 2004), even less studies of retinal damage have been developed in squamate reptiles (Ávila-Mendoza et al., 2016), and the regenerative response following acute retinal injury has remained unexplored. Here, we show that, similarly to other vertebrates, intravitreal injection of NMDA in bearded dragon specifically ablates cells from both iINL and GCL regions throughout the entire retina. Furthermore, we demonstrate for the first time that although the RCJ growth zone at the retinal margin does not get activated by NMDA treatment, at least two different, successive populations of proliferative cells within the central retina respond to excitotoxic damage: one early population of microglia/macrophages – expected to remove cellular debris based on observed IPL thinning previously linked to the destruction and removal of neurites of amacrine cells (Dvorak and Morgan, 1983; Fischer et al., 1998) – and one local population of reactive MG that dedifferentiate to re-express progenitor markers (Figure 7). Importantly, this dual activation pattern of retinal cells has already been described in mammals (Ooto et al., 2004), fish (Braisted et al., 1994; Vihtelic and Hyde, 2000; Fimbel et al., 2007; Nagashima et al., 2013), and birds (Fischer et al., 1998; Fischer and Reh, 2001), thus highlighting the major importance of glia for retinal regeneration across vertebrates (Figure 7).

### Response of Peripheral Retina to Acute Damage

Our previous study revealed that squamates such as bearded dragon lizards possess a proliferative peripheral CMZ-like or RCJ region that acts as a source of retinal progenitors contributing to postnatal ocular growth (Eymann et al., 2019). Our new findings now fail to indicate any significant increase of proliferation at the RCJ, making it unlikely that this region contributes to neuronal regeneration in response to neurotoxin-induced retinal injury (Figure 7). Although the proliferative response was not assessed in a different model of severe excitotoxic retinal injury (Ávila-Mendoza et al., 2016), a similar lack of RCJ response was noticed upon optic nerve lesions involving relatively low retinal cell death (Beazley et al., 1998; Lang et al., 2002; Dunlop et al., 2004). Successful, spontaneous CMZ-mediated retinal regeneration has only been documented in fish and amphibians in response to injuries including peripheral retina damage and in some cases of full retinal ablation (Figure 7, left panels), and long-distance migration of progenitor cells within injured or diseased retina has never been observed in vertebrates. In *Xenopus*, mechanical retinectomy and transgenic rod ablation induce a CMZ response in form of increased proliferation, expanded size, and neurogenesis (Yoshii et al., 2007; Choi et al., 2011; Miyake and Araki, 2014; Langhe et al., 2017). In fish, the regenerative response at the CMZ has received less attention, but few existing reports are in line with the observations in frogs. In medaka, for example, needle puncture of the peripheral retina stimulates the CMZ’s proliferative progenitor pool, resulting in a short burst of accelerated peripheral neurogenesis in response to local damage (Centanin et al., 2014). Similarly, neurotoxic damage in the goldfish retina leads to increase of both CMZ proliferative activity and *de novo* generation of neurons at the retinal periphery (Maier and Wolburg, 1979; Negishi et al., 1982; Raymond et al., 1988; Stenkamp et al., 2001). In contrast, the CMZ or retinal margin in mammals and birds shows a reduced regenerative capacity (Fischer and Reh, 2000; Moshiri and Reh, 2004; Jian et al., 2009; Kiyama et al., 2012), which is reminiscent of their very limited postnatal neurogenic capacity under physiological conditions (Kubota et al., 2002). Nevertheless, activation of retinal margin proliferation has been reported to some extent in transgenic mammalian models of continuous ganglion cell (Kiyama et al., 2012) or photoreceptor degeneration (Moshiri and Reh, 2004; Jian et al., 2009). However, regenerative neurogenesis and replenishment processes in these transgenic models usually decline with age (Jian et al., 2009), require additional modifications of signaling pathways (Moshiri and Reh, 2004), and/or depend on other factors such as damage severity (Kiyama et al., 2012). Similarly, despite the persistence of CMZ progenitors in young postnatal chicks (Kubota et al., 2002), acute retinal damage alone without administration of exogenous growth factors does not stimulate regenerative activity at the retinal margin in this bird model (Fischer and Reh, 2000; Fischer, 2005). Based on these previous amniote studies, it is thus conceivable that progenitors previously identified in the bearded dragon RCJ might only contribute to peripheral retina regeneration when stimulated by growth factors. Alternatively, specific differences in the ability for retinal regeneration might exist among squamates, as different lizard and snake species are already known to exhibit significant variation in both their postnatal RCJ proliferative activity (Eymann et al., 2019) and regenerative capacity following optic nerve damage (Beazley et al., 1998; Lang et al., 2002; Dunlop et al., 2004). In bearded dragon lizards, our previous findings clearly indicate the maintenance of a source of highly proliferating RCJ progenitors contributing to ocular growth throughout adulthood (Eymann et al., 2019). In this growth context, the bearded dragon RCJ might rather display a dynamic proliferative activity in response to conditions affecting eye size, including form-deprivation myopia and/or nutritional changes.

### Activation of Glial Cells in Response to Acute Retinal Damage

Beyond the peripheral margin, regeneration in the central retina has been the focus of many research efforts, and several cellular sources for neural regeneration have been characterized in vertebrates (Figure 7, right panels). Spontaneous regeneration of the central retina in response to acute damage is best studied in fish, where MG cells have been identified as the main source of regeneration in response to various tested damage paradigms ranging from mechanical wounds (Fausett and Goldman, 2006), light lesions (Bernardos et al., 2007), and neurotoxic ablation (Fimbel et al., 2007) to transgenic degenerative models (Figure 7). In these studies, each specific damage usually affects different retinal cell populations and/or triggers variable regenerative efficiencies, with outer retinal neurons such as rod and cone photoreceptors usually showing a faster and higher regenerative success than innermost retinal neurons. Nonetheless, MG activation shares common hallmarks (Nagashima et al., 2013; Powell et al., 2016) such as dedifferentiation, proliferation and production of clusters of MGDPs in the INL, as well as migration of MGDPs to other retinal layers and differentiation into neurons (Figure 7). In amphibians, MG-mediated retinal regeneration has been rarely analyzed, presumably due to the prevalence of studies applying full mechanical retinectomy and therefore removing MG cells. In these cases, regeneration of the entire retina is well-established to occur via transdifferentiation of RPE cells (Negishi et al., 1992; Yoshii et al., 2007). Nevertheless, similarly to the fish situation, a few reports using less drastic damage paradigms have observed MG reactivation involving dedifferentiation with marked expression of progenitor markers, cell cycle re-entry, and regeneration of retinal neurons (Langhe et al., 2017; Hamon et al., 2019). In birds, the regenerative capacity of MG in response to acute damage is strongly reduced and limited to newborn chicks, as it progressively disappears from central retina to be restricted to more peripheral areas already during the first postnatal month (Fischer and Reh, 2002, 2003b). Furthermore, MG dedifferentiation and proliferation processes are only induced following treatment with high doses of neurotoxin treatment (Fischer and Reh, 2001), and the majority of cells generated by proliferating MG remain as undifferentiated progenitor-like cells unless stimulated by exogenous growth factor cocktails (Fischer and Reh, 2002). In mammals, the regenerative response is yet more limited and strictly dependent on stimulation with growth factors (Close et al., 2006; Karl et al., 2008; Jorstad et al., 2017) and/or transgenic reprogramming of MG (Figure 7; Ueki et al., 2015; Jorstad et al., 2017; Hamon et al., 2019; Rueda et al., 2019).

In squamates, previous studies employing optic nerve lesions suggested that neurogenesis is likely absent in the naïve and recovering central retina, but the relatively low rate of retinal ganglion cell degeneration induced by these models have likely hampered any proliferative response (Beazley et al., 1998; Lang et al., 2002; Dunlop et al., 2004). Similarly to other anamniote and amniote species tested so far, our new study demonstrates the reactivation of bearded dragon MG in response to acute retinal damage. The regenerative response in our lizard model includes MG proliferation and dedifferentiation as well as some degree of MGDP migration and proneural specification, indicating that the endogenous capacity of MG to become retinal progenitor is common to all vertebrate groups (Figure 7). Importantly, however, the regenerative capacity of MG is highly variable between vertebrate species, and our findings reveal that squamate reptiles lie between fish/amphibians and mammals/birds in the spectrum of retinal regeneration. For example, the proliferative expansion and clustering of MGDPs observed in bearded dragon are not as robust as in fish (Fausett and Goldman, 2006; Bernardos et al., 2007; Thummel et al., 2008). In contrast, despite the delayed activation of MG in response to similar NMDA dose, the overall regenerative capacity at central retina in our juvenile lizards appears increased in terms of MGDP proliferation and differentiation patterns, when compared to the age-dependent response restricted to hatchling stages in chicken (Fischer and Reh, 2002, 2003a). However, squamates are already known to exhibit significant variation in both retinal progenitor activity (Eymann et al., 2019) and regenerative capacity of retinal ganglion cells following optic nerve injury (Beazley et al., 1998; Lang et al., 2002; Dunlop et al., 2004), indicating that different lizard and/or snake species might indeed have different capacities for retinal regeneration. Furthermore, we suspect that the observed delayed apoptotic and regenerative responses in our bearded dragon model may be due to heterogeneity in the time course of retinal regeneration among squamate species. The responses may even vary within the same species depending on the animal age (only juveniles were tested in this study) and/or experimental damage. In fact, a similar situation already exists in other vertebrate models such as zebrafish, where microglia and MG respond to acute light lesion or neurotoxic ablation of inner retinal neurons within a few hours or days, respectively (Nagashima et al., 2013). Based on previous vertebrate findings showing that activation of microglia/macrophages is a key step in stimulating MG progenitor phenotype and cell cycle re-entry (Fischer et al., 2014; Conedera et al., 2019), we further expect that the initial delayed time course of microglia/macrophage recruitment impacts subsequent MG activation in squamates. In line with this hypothesis, the first proliferative MG cells are detected at 9 dpi in our lizard model, a timing corresponding to the microglial invasion of more apical strata of the INL where MG cells reside, suggesting that MG activation might depend on interaction with the microglia/macrophage population. However, the activation and fate of proliferative glial cell populations in the squamate retina after injury remain a topic for future investigation.

## Conclusion

In conclusion, our NMDA-induced retinal damage in bearded dragon reveals for the first time that squamates hold the potential for retinal regeneration though activation of two populations of glial cells, including an endogenous source of MG, in central retina. These new findings further highlight the major importance of glia for retinal regeneration and indicate that squamates lie between anamniote and other amniote groups in their regenerative capacities, thus helping to complete our evolutionary understanding of tissue regeneration in vertebrates. Furthermore, such new understanding of self-repair mechanisms in non-classical models endowed with regenerative properties may help designing therapeutic strategies to human patients or animal models with retinal diseases.

## Data Availability Statement

All datasets generated for this study are included in the article/Supplementary Material.

## Ethics Statement

All reptile captive breedings and experiments were approved by the Laboratory Animal Centre (LAC) of the University of Helsinki and/or the National Animal Experiment Board (ELLA) in Finland (license numbers ESLH-2007-07445/ym-23, ESAVI/7484/04.10.07/2016, and ESAVI/13139/04.10.05/2017).

## Author Contributions

JE and ND-P designed the experimental approach, performed all the experiments, analyzed the data, and wrote the manuscript. JE collected and prepared the samples. Both authors read and approved the final manuscript.

## Conflict of Interest

The authors declare that the research was conducted in the absence of any commercial or financial relationships that could be construed as a potential conflict of interest.
