# Supplementary material for: Glia-Mediated Regenerative Response Following Acute Excitotoxic Damage in the Postnatal Squamate Retina
Source: Front Cell Dev Biol. 2020 May 28;8:406. doi: 10.3389/fcell.2020.00406 (PMC7270358; doi:10.3389/fcell.2020.00406)
Supplement: Supplementary file 1 [file Data_Sheet_1.PDF]

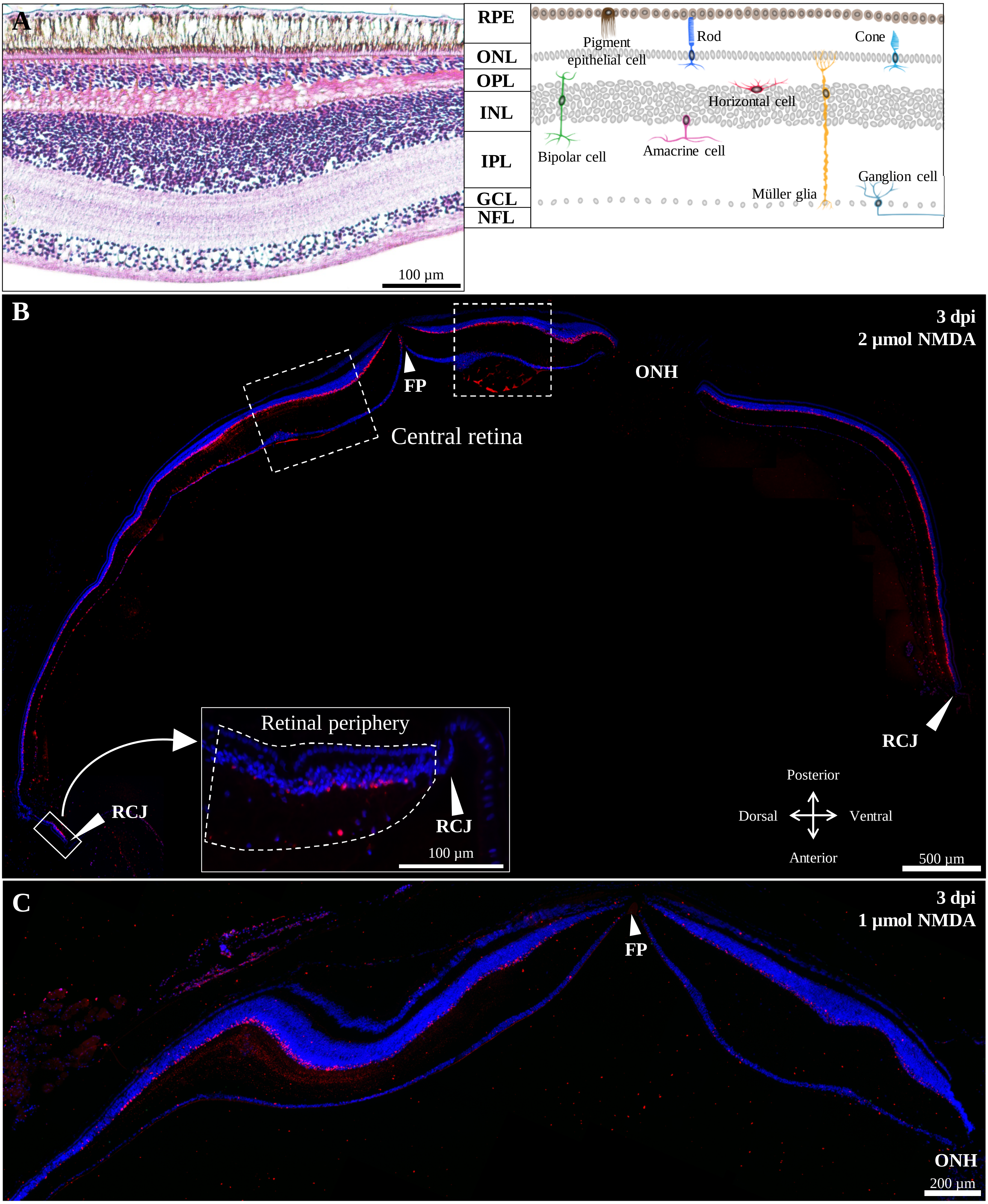

Supplementary Figure S1. (A) Hematoxylin and Eosin histological staining (left) and schematic representation (right) of central retina morphology in bearded dragon. The different layers (RPE, retinal pigment epithelium; ONL, outer nuclear layer; OPL, outer plexiform layer; INL, inner nuclear layer; IPL, inner plexiform layer; GCL, ganglion cell layer; NFL, nerve fiber layer) and cell types are indicated. **(B,C)** TUNEL apoptotic assay (red staining) in the retina of bearded dragon eyes treated with 2 **(B)** or 1 **(C)** µmol of NMDA at 3 days post-injection (dpi). The positions of the pseudostratified retinociliary junction (RCJ), foveal pit (FP), and optic nerve head (ONH) are shown. Insets indicate the central and peripheral retina areas used for cell quantification.
